# The Emotional Recession: global declines in emotional intelligence and its impact on organizational retention, burnout, and workforce resilience

**DOI:** 10.3389/fpsyg.2025.1701703

**Published:** 2025-11-12

**Authors:** Joshua M. Freedman, Patricia E. Freedman, Daniel Y. Choi, Michael Miller

**Affiliations:** Six Seconds, Freedom, CA, United States

**Keywords:** emotional intelligence, decline in emotional intelligence, workplace wellbeing, employee burnout, employee engagement, organizational resilience, retention, organizational performance

## Abstract

**Introduction:**

The COVID-19 pandemic triggered widespread disruptions to workplace wellbeing and employee engagement, intensifying risks of burnout and reduced retention. Although organizational performance has been widely studied, few investigations have examined longitudinal declines in emotional intelligence (EQ) during the pandemic and its aftermath or considered their implications for organizational resilience.

**Methods:**

This study analyzed EQ trends from 2019 to 2024 using data from the Six Seconds Emotional Intelligence Assessment (SEI), a validated 77-item instrument measuring eight core competencies and four life outcome factors. The stratified sample comprised 28,000 adults across 166 countries.

**Results:**

Global EQ scores declined by 5.79% (Cohen’s *d* = 0.22, 95% CI [0.17, 0.27]), with statistically significant decreases across all eight competencies (*p* < 0.001). The largest declines occurred in Drive-related skills, including Engage Intrinsic Motivation (Cohen’s *d* = 0.22, 95% CI [0.17, 0.27]), Exercise Optimism (Cohen’s *d* = 0.23, 95% CI [0.18, 0.28]), and Pursue Noble Goals (Cohen’s *d* = 0.19, 95% CI [0.14, 0.24]). Success Factors—Effectiveness (Cohen’s *d* = 0.20, 95% CI [0.15, 0.25]), Relationships (Cohen’s *d* = 0.19, 95% CI [0.14, 0.24]), Quality of Life (Cohen’s *d* = 0.17, 95% CI [0.12, 0.22]), and Wellbeing (Cohen’s *d* = 0.13, 95% CI [0.08, 0.18])—declined by 4.39–6.45%. Individuals with higher EQ were 10.18 times more likely to report strong overall life outcomes (OR = 10.18, 95% CI [9.64, 10.76]).

**Discussion:**

These findings reveal an “Emotional Recession,” a sustained global decline in emotional and relational capacities with direct implications for wellbeing, engagement, and organizational resilience.

## 1 Introduction

The COVID-19 pandemic disrupted daily life and work around the world, triggering sustained stress, uncertainty, and emotional strain (World Health Organization, 2022; Kniffin et al., 2021). Emotional intelligence (EQ) has been defined in various ways since Salovey and Mayer first described it as the ability to perceive, understand, and manage emotions (Salovey and Mayer, 1990). In this paper however, EQ is understood as a set of learnable emotional and cognitive capacities that enable people to integrate thinking and feeling to make optimal decisions (Freedman, 2010, 2012). Understanding emotional intelligence as a learnable set of skills provides a framework for analyzing how people and organizations adapt to sustained stress and uncertainty, which were intensified during the COVID-19 pandemic. This framing reflects a conceptualization of EQ as a practical, developable capability expressed through self-awareness, intentional action, and contribution to shared purpose. The Six Seconds Emotional Intelligence Assessment (SEI) operationalizes this mixed-model framework through self-report measures of self-perceived emotional skills and attitudes, providing insight into how individuals apply emotional intelligence in everyday life and work.

Building on this conceptual foundation, prior research demonstrates how these emotional capacities influence organizational functioning. In the face of disruption and prolonged uncertainty, emotional intelligence enables people and organizations to adapt, recover, and perform effectively. Higher EQ supports stronger problem-solving, stress regulation, communication, and leadership (O’Boyle et al., 2011). At the interpersonal and organizational level, these capabilities foster trust, collaboration, and cohesion that sustain resilient workplace cultures (Druskat and Wolff, 2001; Bass and Riggio, 2023). Emotional intelligence also helps buffer stress and maintain wellbeing in volatile conditions (Kotsou et al., 2019; Doğru, 2022), supporting adaptive performance in complex work environments (Sharma, 2024; Nguyen et al., 2023). Together, these findings position EQ as a vital resource for sustaining psychological health and organizational effectiveness in demanding contexts. Yet during the pandemic, the very conditions that make EQ essential were severely tested.

The pandemic produced widespread increases in anxiety, burnout, and social disconnection (Killgore et al., 2020; World Health Organization, 2024), with global rates of anxiety and depression rising by 25% in 2020 alone (World Health Organization, 2022). Complementing these global estimates, a UK longitudinal probability sample documented significant increases in psychological distress during 2020 compared with prior years (Pierce et al., 2020). These psychological effects, further exacerbated by shifting work routines, economic insecurity, and prolonged uncertainty (Kniffin et al., 2021), contributed to the global burden of anxiety and depression quantified by Santomauro et al. (2021). Despite extensive documentation of the pandemic’s psychological impacts (Pfefferbaum and North, 2020; Vindegaard and Benros, 2020; Salari et al., 2020), less is known about how emotional capacities have shifted over time. Few studies have examined longitudinal changes in emotional intelligence (EQ) or considered how reductions in emotional capacity weaken individual coping with stress and uncertainty and the collective resources that sustain workplace culture and organizational cohesion.

This study responds to that gap in research by examining two primary objectives. The first was to examine global changes in emotional intelligence (EQ) from 2019 to 2024, framing these declines as an “Emotional Recession” with implications for organizational performance, workforce wellbeing, and resilience.

The second was to investigate patterns within the Six Seconds Emotional Intelligence (SEI) framework, with particular focus on the consistently low scores observed in the Wellbeing Success Factor and the sharp decreases in the EQ competencies comprising the Drive strand. Guided by the Job Demands–Resources model and prior evidence linking EQ to workplace outcomes, it was expected that lower Drive strand competencies would be associated with greater burnout risk, and that individuals with higher EQ would be more likely to report strong outcomes across the SEI Success Factors overall. The study aimed to determine whether individuals with higher EQ would show markedly greater odds of scoring in the top range on all four Success Factors, thereby providing evidence for EQ as a high-leverage organizational asset. These objectives informed the analyses and provided the structure for the key findings presented in Sections 4.1–4.4.

To investigate these objectives, we analyzed EQ trends from 2019 to 2024 using data from the Six Seconds Emotional Intelligence Assessment (SEI), a validated measure applied globally in educational, corporate, and public health settings (Dijk and Freedman, 2007; Emmerling, 2021). The stratified sample includes 28,000 adults across 166 countries, enabling year-over-year analysis of EQ competencies and their relationships to organizational outcomes, with particular attention to how changes in these competencies may reflect and influence post-pandemic workplace culture.

We introduce the concept of an “Emotional Recession” to provide a unifying lens for interpreting these findings. An Emotional Recession is defined as a sustained decline in emotional and relational capacities with measurable consequences for wellbeing and organizational functioning (Freedman et al., 2024). This framework draws on the economic definition of “recession” as a prolonged downturn in key performance indicators (National Bureau of Economic Research, 2020; International Monetary Fund, 2023), the Emotional Recession describes a parallel erosion in emotional intelligence skills essential for social and organizational health: intrinsic motivation, trust, engagement in quality relationships, and resilience against burnout. This contraction of emotional capacity weakens workplace cultures by reducing the energy, empathy, and collaboration resources available. Declines in trust and shared purpose increase the risk of disengagement and turnover.

Within the Emotional Recession framework, the study examines how EQ interacts with four outcomes measured by the SEI: Effectiveness, Relationships, Quality of Life, and Wellbeing. Known as Success Factors, these outcomes reflect the broader organizational impact of emotional intelligence. Wellbeing, defined in the SEI model as the capacity to sustain optimal energy and functioning, was the lowest-scoring outcome.

To make sense of these shifts, we draw on the Job Demands–Resources (JD-R) model developed by Bakker and Demerouti (2007), a foundational theory in organizational psychology that has since been extended to crisis contexts, making it particularly relevant to the COVID-19 period analyzed in this study (Bakker, 2023; Demerouti and Bakker, 2023). The JD-R model links burnout to an imbalance between demands and resources, clarifying how declining emotional intelligence competencies undermine wellbeing, performance, and the collaborative culture needed for engagement (Schaufeli and Taris, 2014). Within this framework, reduced optimism and intrinsic motivation represent a depletion of personal resources, diminishing employees’ capacity to meet job demands and heightening the risk of disengagement, turnover, and erosion of shared purpose. Because the JD-R framework is intentionally broad, specifying context-relevant resources such as EQ is essential for understanding how emotional decline translates into organizational risk.

We also examine the roles of psychological safety, affective commitment, and employee engagement—constructs that help explain how declining EQ translates into organizational risk and culture change. Edmondson (1999) defines psychological safety as a shared belief that the team is safe for interpersonal risk-taking, a condition foundational for open communication and collaboration (Frazier et al., 2017). Affective commitment reflects employees’ emotional attachment to their organization (Meyer and Allen, 1991), a factor closely tied to workforce cohesion and retention. Engagement, characterized by vigor, dedication, and absorption in work, depends on sustained emotional energy and a sense of shared purpose (Schaufeli et al., 2002; Knight et al., 2017). Declines in EQ competencies weaken these workplace culture elements, as reflected in declines in Relationships and Wellbeing factors. Together, these mechanisms illustrate how reductions in EQ contribute to broader patterns of organizational strain consistent with the emerging framework of an Emotional Recession.

## 2 Materials and methods

### 2.1 Participants

This study analyzed data from 28,000 adult participants drawn from the larger 276,972 participant global database of the Six Seconds Emotional Intelligence Assessment (SEI), a 77-item self-report instrument (Emmerling, 2021; Freedman et al., 2005) available in more than 22 languages and used in over 169 countries. Data for this study were collected between 2019 and 2024 in applied settings and subsequently aggregated for research. A stratified random sampling method was used to ensure balanced representation across global region, age group, and gender. Equal numbers of participants were selected from each of seven global regions, defined using continental boundaries from MapsOfWorld.com (n.d.), which the SEI instrument employs for stratification. These regions broadly align with the United Nations M49 statistical classification for continental regions (United Nations Statistics Division, 2023), providing a conceptual benchmark for standard regional grouping. Within each region, sampling achieved equal representation of individuals over and under age 40 and balanced proportions of male and female participants. While the SEI includes a non-binary gender option, responses in this category were excluded due to insufficient sample size for valid comparison.

The final sample comprised 14,000 male (*M* = 39.48, SD = 10.76) and 14,000 female (*M* = 39.22, SD = 10.97) participants. Table 1 summarizes the demographic characteristics of the sample, including gender and age distribution.

**Table 1:** SEI samples’ descriptive statistics.

Gender	*N* participants	Age (*M, SD*)
Female	14,000	39.22 (10.97)
Male	14,000	39.48 (10.76)

Descriptive statistics for the SEI sample across 2019–2024, including total N, mean age, and gender distribution.

### 2.2 Procedure

Participants completed the SEI as part of applied practice settings, including training, coaching, and organizational development programs. Prior to accessing the assessment, all individuals were required to review and accept the instrument’s terms of use and privacy policy, which stated that responses may be stored in anonymized form for future research. These terms align with international data protection standards, including Regulation (EU) 2016/679, the General Data Protection Regulation (GDPR) (European Parliament and Council, 2016).

All response data were collected through a secure digital platform. Personally identifiable information was either never collected or was removed during processing to ensure complete anonymization. For this study, de-identified records were extracted retrospectively and aggregated for analysis without any access to individual-level identifiers or direct participant contact.

Dataset preparation followed a standardized preprocessing protocol, which included excluding test entries, statistical outliers, and records missing essential demographic variables needed for stratified sampling. Stratification and demographic balancing procedures are detailed in Section 2.1 (Participants).

### 2.3 Materials

The Six Seconds Emotional Intelligence Assessment (SEI) is a 77-item self-report instrument designed to measure key dimensions of internal competencies and self-perceived life outcomes associated with emotional intelligence. The SEI is widely used in coaching, leadership development, and organizational training, making it a relevant tool for assessing emotional intelligence in workplace settings. Items are distributed across eight emotional intelligence competencies that form the foundation of the Six Seconds Model of Emotional Intelligence, four outcome factors, and additional demographic questions at the beginning of the assessment.

Respondents rate items using a five-point Likert scale ranging from “strongly disagree” to “strongly agree.” Items reflect emotional and behavioral patterns, such as “If I wanted to, I could tell a friend how I usually react to stress,” “I have many people that I can fully rely on,” “I am able to predict my reactions,” “There is a logic to feelings,” and “I’ve effectively resolved challenges.” Items listed are paraphrased examples.

Table 2 presents the eight SEI competencies and their definitions. Each competency represents a distinct emotional intelligence skill and is scored individually (Stillman et al., 2017). The competencies are further grouped into developmental strands that capture related capabilities, as documented in the SEI technical manual (Freedman et al., 2023). One such strand, Drive, reflects future orientation and a sense of agency, and is composed of three competencies: Exercise Optimism, Engage Intrinsic Motivation, and Pursue Noble Goals.

**Table 2:** SEI competencies and definitions.

Competency	Definition
EEL: Enhance emotional literacy	Accurately identifying and interpreting both simple and compound feelings
RP: Recognize Patterns	Acknowledging frequently recurring reactions and behaviors
ACT: Apply consequential thinking	Evaluating the costs and benefits of your choices
NE: Navigate emotions	Assessing, harnessing, and transforming emotions as a strategic resource
EIM: Engage intrinsic motivation	Gaining energy from personal values & commitments vs. being driven by external forces
EO: Exercise optimism	Taking a proactive perspective of hope and possibility
IE: Increase empathy	Recognizing and appropriately responding to others’ emotions
NG: PNG: Pursue noble goals	Connecting your daily choices with your overarching sense of purpose

The eight SEI emotional intelligence competencies and their brief definitions: Enhance Emotional Literacy, Recognize Patterns, Apply Consequential Thinking, Navigate Emotions, Engage Intrinsic Motivation, Exercise Optimism, Increase Empathy, and Pursue Noble Goals.

In addition to competencies, the SEI yields scores on four self-reported life outcomes, referred to as Success Factors: Effectiveness, Relationships, Quality of Life, and Wellbeing. Each Success Factor consists of two subscales. Effectiveness includes Influence and Decision Making; Relationships includes Network and Community; Quality of Life includes Achievement and Satisfaction; and Wellbeing includes Balance and Health. Table 3 provides definitions for the four Success Factors. Together, the competencies and Success Factors allow for a contextualized interpretation of emotional intelligence, linking internal capacities to external indicators of functioning (Freedman et al., 2019).

**Table 3:** SEI success factors and definitions.

Success factor	Definition
Overall outcomes	Average of the four success factors
Effectiveness	Capacity to generate results
Relationships	Capacity to build and maintain networks
Wellbeing	Capacity to maintain optimal energy and functioning
Quality of Life	Capacity to maintain balance and satisfaction

Definitions of the four SEI Success Factors used as outcome variables in this study: Wellbeing, Effectiveness, Relationships, and Quality of Life.

The SEI has been psychometrically validated and is used in over 22 languages across 169 countries. It has been applied in business, education, healthcare, military, and nonprofit sectors. Validation studies confirm adequate construct validity for both competency and outcome scales (Stillman et al., 2017; Freedman et al., 2023). Internal consistency for the present dataset was satisfactory, with Cronbach’s *α* coefficients ranging from 0.64 to 0.81 across the SEI scales, consistent with previously published validation (Dijk and Freedman, 2007; Emmerling, 2021).

### 2.4 Data analysis

Quantitative data were drawn from repeated cross-sectional samples from 2019 to 2024 using the SEI. We calculated descriptive statistics for our EQ and outcome measures across the six-year dataset to confirm distributional assumptions and prepare for MANOVA and ANOVA testing. Multivariate Analysis of Variance (MANOVA) followed by univariate tests was then used to examine whether respondents in our sample differed in emotional intelligence and Success Factors over time. Percent change was computed across the six-year period (2019–2024) by comparing average EQ scores from 2019 and 2024.

We analyzed trends over time through MANOVAs, one-way Analysis of Variance (ANOVAs), and post-hoc Tukey’s HSD. MANOVA was selected to control for relationships between dependent variables and reduce the risk of type 1 errors, while ANOVA identified which variables changed and how they changed. By testing all dependent variables simultaneously, MANOVA controls the familywise error rate by reducing the number of separate significance tests, thereby minimizing the likelihood of Type I error inflation. Post-hoc comparisons using Tukey’s HSD identified changes at different points in time.

In order to better understand the relationship between overall EQ and success factor scores, we calculated an odds ratio. Percent change was computed across the six-year period (2019–2024) by comparing average EQ scores from the earliest and most recent data points.

The purpose of an odds ratio is to summarize the strength of the relationship between a variable of interest and an outcome. An odds ratio quantifies how much more likely an outcome is in one group compared to another (Norton et al., 2018). An OR greater than 1 indicates higher odds, and the further above 1, the stronger the association. A very high OR (such as approximately 10) suggests a strong relationship, indicating that individuals in the high-EQ group have markedly higher odds of strong life outcomes compared to those in the low-EQ group. However, this does not imply causality or that the effect is uniform across individuals (Norton et al., 2018).

SEI scores are standardized with a normative mean of 100 and a standard deviation of 15. Although EQ scores were primarily treated as continuous variables, a categorical threshold was required for odds ratio computation. Participants scoring above the sample mean were classified as “high EQ,” while those below the mean were classified as “low EQ.” This approach reflects the expectation that individuals scoring above the mean are more likely to effectively apply their emotional intelligence competencies, whereas those below the mean may experience greater difficulty doing so.

Once we categorized high EQ score–high Outcome score and low EQ score–low Outcome score conditions, we also categorized high EQ score–low Outcome score and low EQ score–high Outcome score conditions prior to calculating the odds ratio (continuous analyses of EQ scores produced comparable associations).

All statistical analyses, including Cronbach’s alpha, MANOVA, ANOVA, Tukey’s HSD, and confidence intervals, were conducted using R (version 4.1.0; R Core Team, 2021).

### 2.5 Ethics approval

This study was approved by the Heartland Institutional Review Board (HIRB project No. 616-102224). Informed consent was obtained from all participants in accordance with institutional ethical standards. The data were originally collected through the SEI questionnaire for non-research purposes such as coaching, training, team selection, and personal development. Under U.S. federal regulations, the use of these de-identified educational assessment results qualifies as exempt research per HHS §46.104(d)(2)(i).

## 3 Results

### 3.1 Descriptive statistics, internal consistency, and data distribution

Descriptive statistics for the emotional intelligence competencies are provided in Table 4, and descriptive statistics for the Success Factors are shown in Table 5. All variables demonstrated approximately normal distributions, with skewness values ranging from −0.51 to 0.08, which did not exceed the conventional absolute value of 0.8, and kurtosis values ranging from −1.04 to −0.6. These values indicate no substantial deviations from normality.

**Table 4:** Emotional intelligence competencies (Descriptive Statistics).

Variable	*N*	*M*	*SD*	Median	Skewness	Kurtosis	*SE*
Total EQ	28,000	99.87	9.83	100.27	−0.17	−0.65	0.06
EEL	28,000	99.70	13.68	100.57	−0.20	−0.93	0.08
RP	28,000	100.58	13.10	101.81	−0.33	−0.81	0.08
ACT	28,000	101.59	13.28	102.51	−0.23	−0.73	0.08
NE	28,000	97.13	14.31	97.24	0.08	−1.00	0.09
EIM	28,000	99.50	13.56	100.9	−0.27	−0.99	0.08
EO	28,000	100.58	13.20	101.97	−0.32	−0.82	0.08
IE	28,000	99.67	14.13	100.9	−0.19	−0.96	0.08
PNG	28,000	99.77	14.03	101.42	−0.36	−1.04	0.08

Descriptive statistics for the eight SEI competencies across the 2019–2024 dataset, including mean scores, standard deviations, and sample sizes.

**Table 5:** Success factor outcomes (descriptive statistics).

Variable	*N*	*M*	*SD*	Median	Skewness	Kurtosis	*SE*
Overall Outcomes	28,000	99.70	9.27	100.39	−0.31	−0.60	0.06
Effectiveness	28,000	100.92	12.10	101.14	−0.22	−0.73	0.07
Relationships	28,000	99.70	11.76	101.47	−0.51	−0.81	0.07
Quality of life	28,000	99.88	11.89	101.95	−0.44	−0.81	0.07
Wellbeing	28,000	98.30	11.65	99.38	−0.31	−0.91	0.07

Descriptive statistics for the four SEI Success Factors across the 2019–2024 dataset, including mean scores, standard deviations, and sample sizes.

Furthermore, internal consistency for the eight Emotional Intelligence Competencies (Table 4) and the four Success Factor scales (Table 5) was evaluated using Cronbach’s alpha. The Cronbach’s alpha levels across the eight competencies were as follows: Enhance Emotional Literacy (*α* = 0.73), Recognize Patterns (α = 0.78), Apply Consequential Thinking (α = 0.64), Navigate Emotions (α = 0.75), Engage Intrinsic Motivation (α = 0.75), Exercise Optimism (α = 0.78), Increase Empathy (α = 0.65), and Pursue Noble Goals (α = 0.80). The Cronbach’s alpha levels across the four Success Factors were as follows: Relationships (α = 0.72), Effectiveness (α = 0.81), Quality of Life (α = 0.73), and Wellbeing (α = 0.65).

### 3.2 Global trends in emotional intelligence scores

From 2019 to 2024, global emotional intelligence (EQ) scores declined significantly, with an overall decrease of 5.79% (Cohen’s *d* = 0.22, 95% CI [0.17, 0.27]). As shown in Supplementary Table S1, total EQ and each of the three SEI pursuits (Know Yourself, Choose Yourself, Give Yourself) declined steadily year by year between 2019 and 2024. Furthermore, this trend of declining scores was consistent across all eight competencies measured by the Six Seconds Emotional Intelligence Assessment (SEI): Enhance Emotional Literacy, Recognize Patterns, Apply Consequential Thinking, Navigate Emotions, Engage Intrinsic Motivation, Exercise Optimism, Increase Empathy, and Pursue Noble Goals (Freedman et al., 2024). Results show a six-year decline, reflecting a broader phenomenon referred to as an Emotional Recession (Freedman et al., 2024).

**Figure 1:**
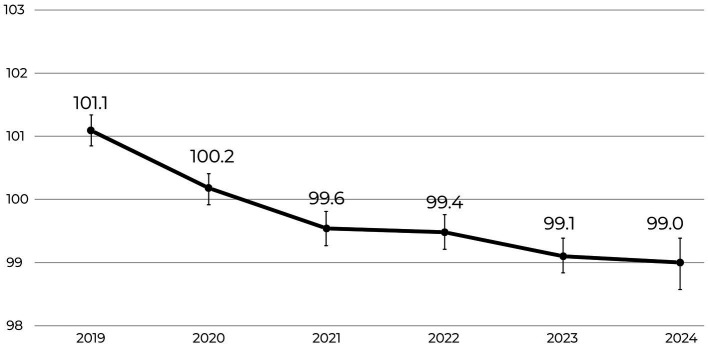
Annual mean emotional intelligence (EQ) scores from 2019 to 2024. Results show a significant decline in EQ over the 6-year period (−5.79%; *p* < 0.001; Cohen’s *d* = 0.22, 95% CI [0.17, 0.27]). Error bars represent 95% confidence intervals.

Figure 1 presents the year-over-year decline in average global EQ scores. A one-way ANOVA revealed a statistically significant effect of year on EQ scores, *F*(1, 27,998) = 134.8, *p* < 0.001, indicating that mean EQ scores differed across the observed six-year period. *Post hoc* comparisons using Tukey’s HSD test indicated that EQ scores declined significantly from 2019 to 2024, with 2024 scores being on average 2.1 points lower than those in 2019 (*p* < 0.001). While differences between subsequent years during the pandemic (2019–2022) were statistically significant, this trend leveled off in later years, suggesting a plateau after the acute disruption of the pandemic.

### 3.3 Emotional intelligence competency-level changes

**Figure 2:**
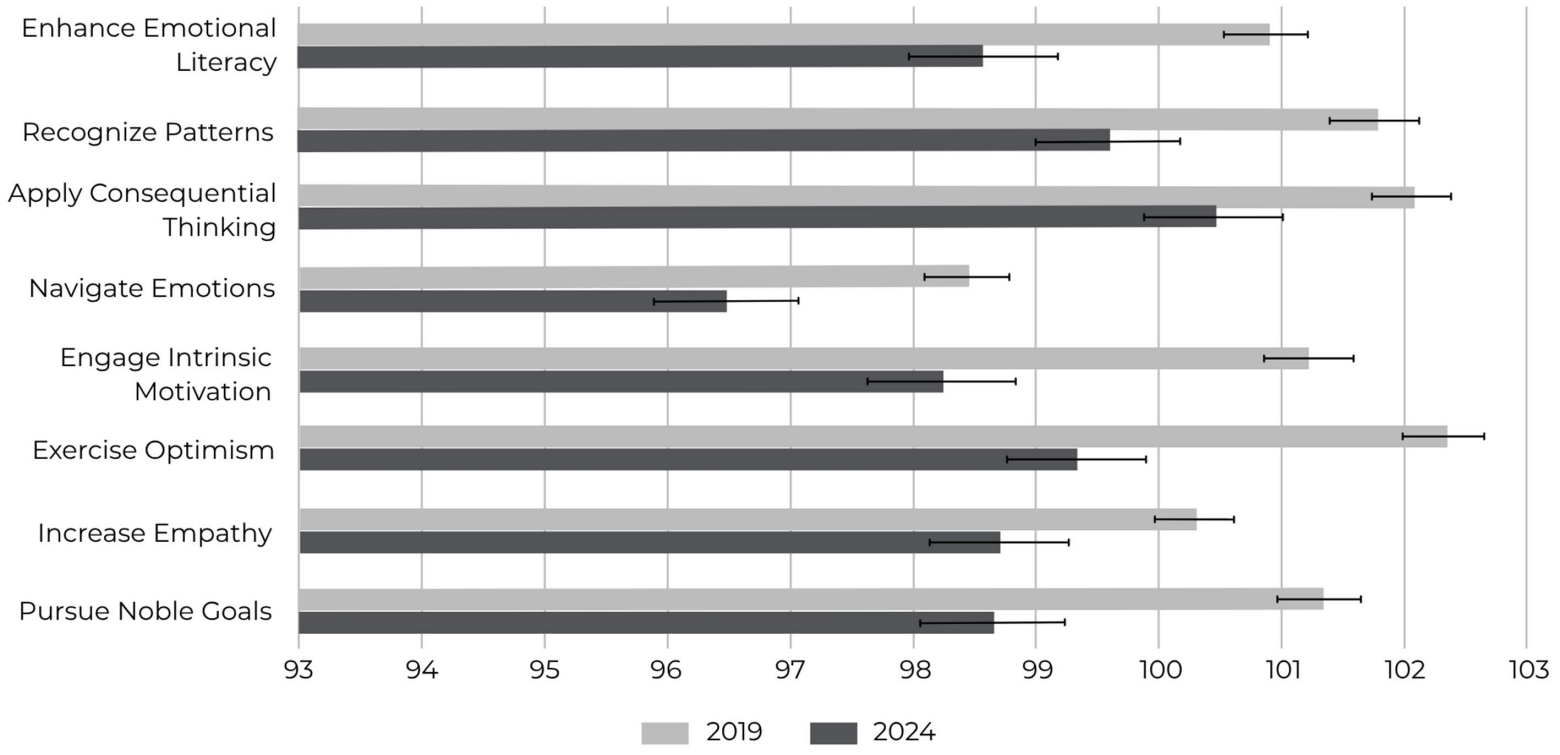
Mean EQ competency scores in 2019 and 2024. All eight competencies declined significantly over time (*p* < 0.001), with small-to-moderate effect sizes (Cohen’s *d* = 0.13–0.23, 95% CIs shown). Error bars represent 95% confidence intervals.

A one-way MANOVA was conducted to examine the effects of time on the eight SEI competency scores. The multivariate analysis using Pillai’s Trace revealed a significant effect of year on EQ competencies, indicating that mean scores differed significantly across years from 2019 to 2024 [Pillai’s Trace = 0.011, *F*(40, 121,995) = 7.42, p < 0.001]. Follow-up univariate ANOVAs and *post hoc* Tukey’s HSD tests identified significant declines across all competencies when comparing 2019 with 2024. Figure 2 illustrates the comparative changes in competency means between 2019 and 2024.

Enhance Emotional Literacy: Mean diff = −2.3, 95% CI [−3.31, −1.33], *p* < 0.001; Recognize Patterns: Mean diff = −2.2, 95% CI [−3.12, −1.23], *p* < 0.001; Apply Consequential Thinking: Mean diff = −1.6, 95% CI [−2.57, −0.65], *p* < 0.001; Navigate Emotions: Mean diff = −2.0, 95% CI [−3.01, −0.94], *p* < 0.001; Engage Intrinsic Motivation: Mean diff = −3.0, 95% CI [−3.95, −2.00], *p* < 0.001; Exercise Optimism: Mean diff = −3.0, 95% CI [−3.95, −2.05], *p* < 0.001; Increase Empathy: Mean diff = −1.6, 95% CI [−2.62, −0.57], *p* < 0.001; Pursue Noble Goals: Mean diff = −2.6, 95% CI [−3.69, −1.66], *p* < 0.001. Detailed annual means for all eight competencies are presented in Supplementary Table S2, which confirms consistent declines across the 6-year span.

**Figure 3:**
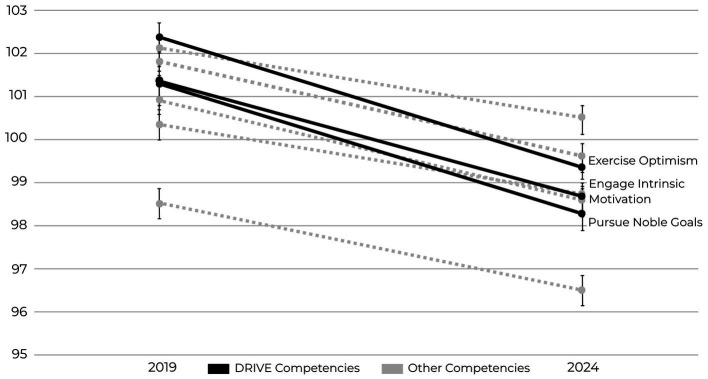
Mean scores for the SEI drive strand competencies (Exercise Optimism, Engage Intrinsic Motivation, Pursue Noble Goals) in 2019 and 2024. Drive competencies showed the steepest declines (−7–8%; *p* < 0.001; Cohen’s *d* ≈ 0.19–0.23, 95% CIs shown). Error bars represent 95% confidence intervals.

To further examine patterns within the Drive strand, Figure 3 shows all eight SEI competency scores in 2019 and 2024, with the three Drive competencies (Exercise Optimism, Engage Intrinsic Motivation, and Pursue Noble Goals) highlighted. These competencies declined more steeply than the others. As shown in Supplementary Table S3, the three Drive competencies declined by 7–8%, compared with smaller 4–6% decreases in the other competencies.

### 3.4 SEI success factor changes

**Figure 4:**
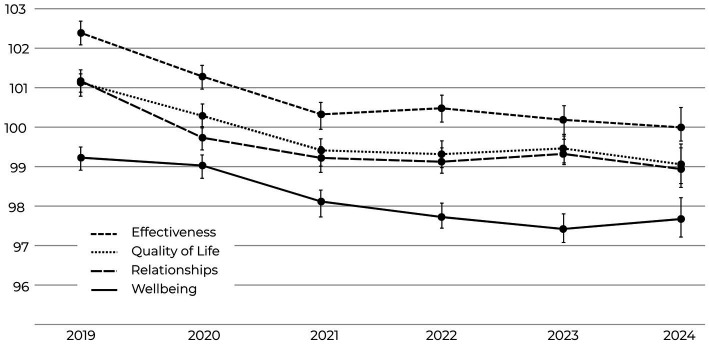
Mean scores for SEI success factors (Wellbeing, Effectiveness, Relationships, and Quality of Life) from 2019 to 2024. All Success Factors declined significantly (−4.39–6.45%; *p* < 0.001), with Wellbeing remaining lowest throughout (Cohen’s *d* = 0.13, 95% CI [0.08, 0.18]). Error bars represent 95% confidence intervals.

There are four SEI Success Factors: Wellbeing, Effectiveness, Relationships, and Quality of Life. In this study, analyses focused on the three most closely tied to organizational performance: Wellbeing, Effectiveness, and Relationships. Analysis of the four Success Factors revealed declines across all outcomes between 2019 and 2024. Relationships and Effectiveness showed the steepest decreases over time (−6.35% – Relationships Cohen’s *d* = 0.19, 95% CI [0.14, 0.24] and −6.15% – Effectiveness Cohen’s *d* = 0.20, 95% CI [0.15, 0.25], respectively), while Wellbeing consistently remained the lowest-scoring outcome factor across all 6 years (−4.97% decline overall – Cohen’s *d* = 0.13, 95% CI [0.08, 0.18]). Figure 4 illustrates the comparative downward trends, and Supplementary Table S4 provides the annual outcome scores along with percentage changes across the six-year timespan.

### 3.5 Emotional intelligence odds ratios for success factors

**Figure 5:**
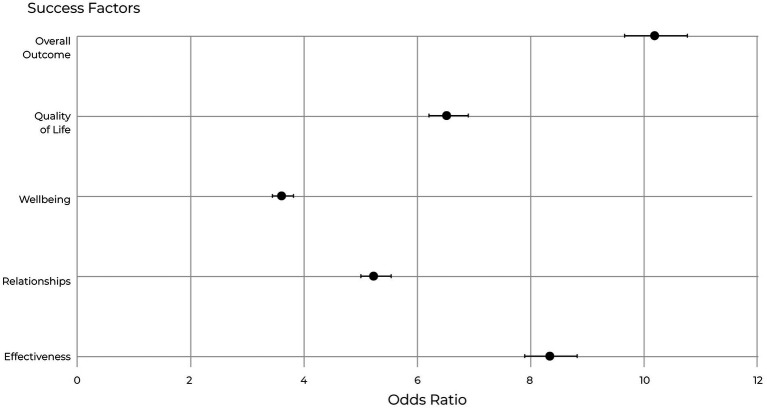
Odds ratios for high EQ and high success factors. Individuals with higher EQ scores were 10.18 times more likely to report strong overall life outcomes across combined success factors (OR = 10.18, 95% CI [9.64, 10.76]).

Individuals that were in the group that scored higher than the average EQ score were more than 10 times more likely to report strong overall outcomes across Effectiveness, Relationships, Quality of Life, and Wellbeing combined (OR = 10.18, 95% CI [9.64, 10.76]). When examined separately, the odds ratios for individual Success Factors ranged from 3.63 for Wellbeing to 8.34 for Effectiveness. Individuals in the high EQ condition therefore had greater likelihood of achieving positive outcomes compared with those in the low EQ condition. Figure 5 illustrates the odds ratios for high EQ predicting high success factors, and Supplementary Table S5 provides the odds ratios with confidence intervals for each outcome.

## 4 Discussion

This study reveals a marked and sustained decline in emotional intelligence (EQ) between 2019 and 2024 across a large global sample. The downward trend spans all eight core emotional intelligence competencies and is accompanied by a corresponding decline in self-reported life outcomes, or SEI Success Factors. The observed declines in EQ competencies and Success Factors suggest the emergence of an Emotional Recession, defined as a sustained decline in emotional and relational capacities (Freedman et al., 2024). This pattern aligns with recent research on post-pandemic workforce trends, which reports deteriorations in social–emotional capacities and engagement, with significant implications for burnout and retention (Sharma, 2024; Knight et al., 2019).

Declining EQ competencies, as demonstrated in our results, suggest heightened vulnerability to burnout, disengagement, and emotional exhaustion, outcomes already well documented in the literature. At the organizational level, these declines strain relational foundations. When key assets such as trust, collaboration, and shared purpose weaken, it becomes more difficult to sustain culture and retain employees. To interpret these patterns, the following sections draw on established constructs in organizational psychology, including psychological safety, affective commitment, and employee engagement, which offer a framework for understanding how reductions in emotional intelligence skills affect both performance and workforce stability.

### 4.1 The emotional recession as a strategic risk for organizational resilience

Between 2019 and 2024, global emotional intelligence (EQ) scores declined by an average of 5.79% (Cohen’s *d* = 0.22, 95% CI [0.17, 0.27]) across all eight competencies measured by the Six Seconds Emotional Intelligence Assessment (SEI). This decline was consistent across regions and demographics, with particularly sharp decreases in Exercise Optimism, Engage Intrinsic Motivation, and Pursue Noble Goals (see Section 3.3). These results demonstrate a measurable shift in skills that support trust, collaboration, and organizational functioning, providing empirical support for the characterization of an Emotional Recession.

From an organizational perspective, declining EQ has been linked to reduced affective commitment, a key driver of retention and discretionary effort (Kuok, 2022). This erosion of commitment contributes to higher turnover risk, as affective bonds are central to workforce stability (Maryati and Astuti, 2024). Engagement similarly depends on emotional energy and psychological connection (Schaufeli et al., 2002). The sharp decline in intrinsic motivation suggests that even highly skilled employees may struggle to sustain performance as EQ erodes (Gagné and Deci, 2005). Over time, these pressures strain workplace culture, lower wellbeing and productivity, and threaten innovation and financial outcomes (De Neve et al., 2024).

Taken together, these findings suggest that the Emotional Recession may represent a structural risk to workforce resilience because declining EQ competencies diminish the personal and relational resources needed to adapt to high job demands. Addressing these risks requires prioritizing EQ skill-building and the early identification of emotional decline. Research shows that cultures which invest in emotional intelligence are better able to maintain engagement and adapt to uncertainty (Masalat et al., 2025). EQ also supports psychological safety, which fosters collaboration and shared problem-solving during periods of volatility (Zhang and Xu, 2024). Strengthening EQ in this way enhances workforce resilience, even under volatile, uncertain, complex, and ambiguous (VUCA) conditions (Bennett and Lemoine, 2014).

Within the Job Demands–Resources framework, the Emotional Recession reflects persistently high demands coupled with reduced personal resources in the form of emotional intelligence skills. Capacities such as optimism, intrinsic motivation, and trust are in short supply, leaving employees more vulnerable to burnout and disengagement. Evidence from EQ-based leadership interventions indicates that targeted development can rebuild these resources and strengthen organizational resilience (Iseppato et al., 2024).

### 4.2 Employee burnout and the decline of drive

This section examines the relationship between burnout and the Drive strand of the Six Seconds Emotional Intelligence Assessment (SEI), which showed the steepest declines from 2019 to 2024 (see Figure 3). Drive is a composite of three competencies: Exercise Optimism, which supports a future-focused outlook; Engage Intrinsic Motivation, which fosters self-direction and sustained effort; and Pursue Noble Goals, which promotes purposeful action (Freedman, 2017; Stillman et al., 2017).

Beyond the SEI framework, organizational psychology research has established how these competencies function as motivational and emotional resources that contribute to resilience and sustained engagement. Optimism supports adaptive coping in adversity (Carver and Scheier, 2014). Intrinsic motivation sustains persistence without external reinforcement (Gagné and Deci, 2005) and has recently been linked to lower burnout in organizational contexts (Mendonça et al., 2023). Purpose strengthens meaning at work, which supports prosocial behavior and engagement (Grant, 2008; Steger et al., 2012). Recent studies confirm that meaningful work continues to play a protective role within the JD–R framework (Allan et al., 2019). Burnout reflects the progressive depletion of these motivational and emotional resources, which can erode both individual capacity and organizational performance (Freedman et al., 2024).

Burnout is evidenced by emotional exhaustion, depersonalization, and a diminished sense of personal accomplishment, which tends to intensify when individuals lack the EQ competencies related to optimism and purpose (Salvagioni et al., 2017). When emotional skills such as a sense of agency and relational supports (like a strong network) are compromised, burnout appears to worsen, creating a cycle in which diminished emotional resources further fuel exhaustion and disengagement (Maslach and Leiter, 2016).

Within the Drive strand, the most pronounced decline has been in Engage Intrinsic Motivation, which by 2024 had become the lowest-scoring of the three Drive competencies. Figure 3 illustrates this pattern, showing that while all eight SEI competencies declined between 2019 and 2024, the Drive strand experienced steeper decreases than the other competencies. Engage Intrinsic Motivation reflects the capacity to take purposeful action without dependence on external rewards (Gagné and Deci, 2005). Lower intrinsic motivation is associated with greater disengagement and reduced persistence in the face of challenges (Gagné and Deci, 2005; Mendonça et al., 2023). Exercise Optimism supports a future-focused outlook that sustains adaptive problem-solving; lower optimism is linked to reduced perception of possibilities and alternatives during adversity, which may undermine resilience (Carver and Scheier, 2014). Pursue Noble Goals anchors action to a larger sense of purpose, providing an ethical and motivational compass. Declines in this competency signal a weakening of connection to long-term meaning, which is closely tied to constructs such as meaningful work and prosocial motivation, both positively associated with employee engagement and organizational citizenship behaviors (Steger et al., 2012; Grant, 2008).

In combination, these competencies supply the motivational and emotional resources that support resilience and sustained engagement. These declines appear to weaken the emotional drivers of engagement and ethical action, leaving employees less equipped to manage demanding environments. Prior studies show that emotional intelligence supports stress regulation and buffers against burnout risk (Schutte et al., 2007; Mikolajczak et al., 2007).

At a broader level, lower emotional intelligence has been linked to diminished capacity for recognizing and addressing early indicators of burnout. Studies show that individuals with lower EQ struggle more with managing stress and providing mutual support in workplaces (Görgens-Ekermans and Brand, 2012; Mendonça et al., 2023). Côté (2014) observed that emotional intelligence enables the recognition and regulation of stress signals; when these skills decline, leaders and employees are less able to intervene effectively, limiting their capacity to support one another. Chronic burnout may further deplete the emotional and psychological resources required for maintaining EQ, reinforcing cycles of disengagement and lower morale (Mikolajczak et al., 2007). Together, these dynamics reduce employees’ adaptive capacity and erode the cultural conditions that sustain engagement.

Recent industry reports highlight high rates of burnout, particularly in healthcare, education, and technology. Mercer’s *Global Talent Trends* (2024) report found that more than 80% of employees are at risk of burnout globally, with the rate rising to 83% in Asia over the past year. Aon’s *Global Wellbeing Survey* (2022–2023) similarly reported that 34% of employees listed burnout (including languishing) among their top five workplace wellbeing concerns, second only to mental health at 41% (Mercer, 2024; Aon, 2023). High burnout levels are consistently linked to greater absenteeism, turnover, and healthcare costs, with direct financial consequences for organizations (Nagle et al., 2024). These industry findings parallel our evidence of declining Drive competencies, underscoring that burnout risk is visible both in global survey data and in the erosion of EQ resources within organizations. As EQ competencies within the Drive strand weaken, organizations may lose critical capacity to retain, inspire, and support talent, potentially straining the relational foundations of workplace culture and threaten long-term performance and viability.

To mitigate burnout and its impact on the Drive strand, proactive emotional intelligence skill-building represents a promising approach. Emotional intelligence functions as a personal resource, enabling individuals to regulate stress and recover from challenges. EQ-based leadership fosters trust, collaboration, and recognition, all key resources within the JD-R framework that buffer against burnout (Iseppato et al., 2024). Leaders who apply emotional intelligence can strengthen employees’ adaptive coping and self-regulation, and maintain personal drivers such as optimism, intrinsic motivation, and a sense of purpose. These capabilities protect the emotional energy of the Drive strand and reinforce organizational resilience.

Addressing burnout within the context of the Emotional Recession is likely to be important for sustaining retention and performance over time. By prioritizing emotional intelligence and applying JD-R insights, leaders can foster cultures of engagement and resilience that support wellbeing and safeguard organizational health.

### 4.3 Declines of SEI success factors and workplace wellbeing

There are four SEI Success Factors: Wellbeing, Effectiveness, Relationships, and Quality of Life (Freedman et al., 2019). In this study, we focus on the three most closely tied to organizational performance: Wellbeing, Effectiveness, and Relationships.

Wellbeing consistently remained the lowest-scoring outcome factor across all 6 years (−4.97% decline overall; Cohen’s *d* = 0.13, 95% CI [0.08, 0.18]) a trend that is urgent because diminished wellbeing undermines the capacity to sustain performance over time, as seen in Figure 4. Lower levels of wellbeing are consistently linked to reduced employee engagement, higher absenteeism, and greater vulnerability to burnout (Harter et al., 2003; Cohen et al., 2023), weakening the cultural foundations that support collaboration and long-term organizational outcomes.

Effectiveness is also a critical Success Factor influencing engagement. Research shows that employees who feel effective in their roles are more engaged and perform at higher levels (Harter et al., 2003). These outcomes are further strengthened by emotional intelligence skills that support self-regulation and constructive interaction with others (O’Boyle et al., 2011; Doğru, 2022). Clear role expectations also reinforce engagement by fostering a stronger sense of commitment (Gil-García, 2019).

Relationships form another pillar of workplace engagement and are significantly shaped by overall wellbeing. Positive workplace relationships and strong support networks enhance engagement (Mansour, 2024). When these relationships exist within a climate of psychological safety, employees are more willing to contribute innovative ideas and participate more fully in collaborative problem-solving (Edmondson, 2023).

These declines are consistent with evidence that lower workplace wellbeing is associated with significant economic costs. The *Thriving Workplaces* report (McKinsey Health Institute, 2025) estimates that structuring workplaces to support holistic health could unlock up to US $11.7 trillion globally. Large-scale evidence reinforces the link between employee wellbeing and company performance: firms with higher levels of workplace wellbeing tend to earn greater firm valuations, higher returns on assets, and stronger stock-market performance (De Neve et al., 2024). Organizations that prioritize wellbeing report measurable productivity, engagement, and resilience gains, while those that neglect it face higher turnover, burnout, and erosion of trust in leadership.

Psychological safety is undermined when emotional awareness and regulation are low (Edmondson, 1999). Studies consistently show that teams with lower emotional intelligence struggle to manage conflict and adapt under pressure, a pattern identified in earlier research and confirmed in more recent contexts (Druskat and Wolff, 2001; Gamero et al., 2021). They also find it harder to maintain constructive communication, which undermines collaboration and mutual support — a challenge documented two decades ago and still addressed through current team EQ interventions (Jordan and Troth, 2004; Zhang and Xu, 2024). Declines in EQ are associated with reduced engagement, productivity, and job satisfaction, increasing the risk of burnout and turnover (Ashkanasy and Daus, 2002; Ertiö, 2024).

The Emotional Recession highlights risks to the relational and emotional conditions that enable teams and organizations to function effectively. Given the observed post-pandemic declines, rebuilding Wellbeing, Effectiveness, and Relationships is essential to restoring engagement and resilience. Studies show that perceived wellbeing and employee resilience positively influence work engagement, suggesting that strengthening these Success Factors can deliver measurable returns in improved retention rates and a healthier, more resilient workplace culture (Wut et al., 2022; Robertson and Cooper, 2010).

### 4.4 EQ as a predictor of success and organizational asset

Analysis showed that individuals with higher EQ scores are more than 10 times as likely to report high outcomes in combined Success Factors when compared with those with low EQ (see Figure 5). These findings reinforce that EQ is a strong predictor of outcomes central to organizational functioning.

In practice, higher EQ translates into gains that underpin engagement, retention, and performance (Iseppato et al., 2024). Consistent with these patterns, an EQ development program utilizing the SEI at UCB Pharma reported significant post-intervention gains in teamwork quality and people engagement (Fariselli et al., 2020). Excelitas, an international technology manufacturer specializing in photonic solutions, implemented an EQ-based leadership development program to strengthen engagement and performance across teams. Using the Leadership Vital Signs multi-rater assessment and blended learning approach, 73% of participating leaders demonstrated measurable improvement in leadership effectiveness and engagement (Stillman et al., 2018). These improvements illustrate how targeted EQ interventions can reinforce the workplace factors most closely linked to performance.

Kahn (1990) observed that engagement depends on psychological conditions such as meaningfulness, safety, and availability—conditions that are supported by emotional intelligence competencies. Higher engagement levels are consistently linked to reduced turnover and improved performance. Organizations prioritizing emotional intelligence often experience lower turnover rates and higher employee satisfaction (Suwandana, 2023). Zenger Folkman (2025) found that leaders who regularly demonstrate trust- and empathy-building behaviors, core outcomes of EQ, experience turnover rates 40% lower than their peers, along with higher productivity and satisfaction scores. These findings align with meta-analytic evidence from Miao et al. (2017), which shows that leader emotional intelligence is positively associated with engagement and negatively related to turnover intentions. In periods of economic uncertainty, such relational strengths help preserve connection and cohesion, suggesting that EQ development fortifies the cultural fabric that sustains workforce engagement.

The benefits extend beyond retention to measurable performance gains. Peer-reviewed research confirms that EQ training interventions can lead to sustained improvements in workplace engagement and culture (Mattingly and Kraiger, 2019). For example, EQ training with Siemens Healthineers resulted in a shift in corporate culture, marked by a significant increase in the number of highly engaged leaders and a 46% overall employee engagement score (de Aldan Araujo and Stillman, 2018). Gallup’s Q12 meta-analysis (Gallup, 2024) found that top-quartile engaged teams achieve 23% higher profitability and 18% greater sales productivity than their peers. In a separate analysis, Gallup, Inc (2025) estimated that declining engagement cost the global economy $438 billion in lost productivity in 2024. While many factors influence engagement outcomes, both reports underscore that building emotional competence is a powerful lever for creating the conditions that sustain workforce performance and customer impact.

Recent research supports this perspective. Akaiso (2025) found that higher emotional self-efficacy promotes prosocial behaviors that reinforce organizational coping capacity, enhancing adaptability and performance. A meta-analysis by Powell et al. (2024) demonstrated that EQ competencies can be enhanced through training interventions, leading to sustained improvements in emotional regulation and resilience. These findings reaffirm EQ as a workforce-level asset closely linked to organizational health and long-term adaptability.

Building on this evidence, organizations that integrate emotional intelligence into a systems-level approach through leadership practices, team norms, and cultural systems can amplify the benefits reflected in the 10x odds ratio. When EQ becomes part of the organizational infrastructure, it supports effective leadership, fuels collaboration, and strengthens adaptability. The need for this integration is urgent. Declining EQ competencies erode workforce resilience and weaken performance, particularly during volatile periods. Targeted EQ skill development can restore psychological safety, reduce attrition, and foster cultures of resilience and shared purpose. In an era when disengagement and burnout are undermining capability, rebuilding emotional intelligence is a strategic imperative for sustaining organizational health and long-term success.

### 4.5 Limitations and future directions

This study offers a unique perspective on emotional intelligence (EQ) as a dynamic organizational variable, drawing on a six-year international dataset collected with a validated instrument. However, several limitations should be acknowledged.

The sample may not fully represent the global adult population, as respondents are likely overrepresented in professional roles related to leadership, learning, or human development, with a higher proportion of participants holding post-secondary education. This demographic skew could limit the generalizability of the findings. Future studies would benefit from stratified sampling methods to include a broader range of roles, educational backgrounds, and cultural contexts (Van de Vijver and Leung, 2000; Poortinga and Fontaine, 2022).

The reliance on self-report measures, even with a validated tool (Emmerling, 2021; Freedman et al., 2005), introduces potential biases such as social desirability and limited self-awareness. Recent systematic reviews indicate that some emotional intelligence assessments struggle to demonstrate discriminant validity from personality measures (Bru-Luna et al., 2021; D’Amico and Geraci, 2023). Because this dataset is anonymized, it cannot be correlated with behavioral or physiological indicators. Future research could incorporate complementary methods, including multi-rater tools (e.g., SEI 360), observational assessments, and biometric indicators, to triangulate self-reports and strengthen the construct validity of emotional intelligence as an internal resource (Boyatzis, 2018; Kotsou et al., 2019).

In the context of the SEI specifically, the instrument captures participants’ perceptions of their emotional skills rather than direct assessments of ability or behavior. This approach aligns with mixed-model frameworks that conceptualize EQ as a learnable set of attitudes and capacities applied in daily decision-making. However, self-perception data can be influenced by response style, self-awareness, and situational factors, which may limit the precision with which changes in applied skill can be inferred. As with all self-report measures, SEI scores reflect participants’ perceptions rather than observed behavior, which may introduce self-perception bias. Because both EQ competencies and Success Factors were measured using the same self-report instrument, some common-method variance may also have influenced observed associations.

While internal consistency for the SEI scales was satisfactory overall (Cronbach’s *α* = 0.64–0.81), the minimal detectable change (MDC) for this instrument has not yet been formally established. Two competencies, Apply Consequential Thinking (α = 0.64) and Increase Empathy (α = 0.65), fell slightly below the conventional 0.70 threshold. These coefficients are consistent with prior SEI construct validation and likely reflect the multifaceted nature of these constructs rather than measurement weakness. Consequently, small year-over-year changes in mean EQ scores should be interpreted with caution until future studies confirm the SEI’s sensitivity to change. Continued refinement of these scales and establishment of MDC parameters to strengthen interpretation of longitudinal change are recommended for future research.

While the year-over-year effects on EQ were statistically significant, the multivariate effect size (Pillai’s Trace = 0.011) indicates a modest magnitude of change. This suggests that, although trends are consistent across the large sample, the practical significance at the individual level may be moderate. Future studies could investigate how these small but persistent changes translate into behavioral outcomes over time.

Few longitudinal studies track EQ across multiple years, especially during large-scale disruptions. Multi-wave research is valuable for identifying patterns, predicting outcomes, and evaluating interventions over time (Audulv et al., 2022). This dataset offers a rare perspective on EQ shifts during and after a global crisis, providing a baseline for future organizational research.

The present study provides rare longitudinal evidence of a six-year global decline in emotional intelligence spanning the COVID-19 pandemic and its aftermath. It also introduces the Emotional Recession as a framework for understanding the organizational impact of this decline. The findings demonstrate that EQ functions as both a personal capacity and a collective asset in organizations, shaping wellbeing, collaboration, and adaptability. Addressing this decline requires more than individual skill-building; it calls for an organizational shift that embeds emotional intelligence into leadership, culture, and systemic processes. With evidence showing that higher EQ is associated with more than a tenfold increase in the likelihood of strong outcomes, these results position emotional intelligence as a high-leverage, evidence-based organizational asset for strengthening the workforce’s engagement, resilience, and performance.

## Data Availability

The data analyzed in this study is subject to the following licenses/restrictions: The dataset analyzed in this study is derived from the SEI emotional intelligence assessment, which is the proprietary instrument of Six Seconds. Because of privacy, licensing, and organizational confidentiality restrictions, the raw data cannot be shared publicly. Researchers interested in collaboration or secondary analyses may contact Six Seconds to discuss data access under appropriate agreements. Requests to access these datasets should be directed to daniel.choi@6seconds.org.
