# Supplementary material for: The Emotional Recession: global declines in emotional intelligence and its impact on organizational retention, burnout, and workforce resilience
Source: Front Psychol. 2025 Nov 12;16:1701703. doi: 10.3389/fpsyg.2025.1701703 (PMC12646932; doi:10.3389/fpsyg.2025.1701703)
Supplement: Supplementary file 1 [file Data_Sheet_1.PDF]

Supplementary File 1: Generative AI Prompts and Outputs Used in Manuscript Preparation

Purpose of this Supplementary File
This file documents the use of generative artificial intelligence (ChatGPT-5, OpenAI, August 2025) in the preparation of this manuscript, in compliance with *Frontiers* editorial policy. It includes the prompts submitted to the AI tool and the corresponding outputs that informed sections such as the abstract, discussion, scope statement, and formatting. Representative prompts and outputs are provided below as examples of AI use. Additional exchanges followed the same procedures and were reviewed, edited, and verified by the authors.

All AI-generated material was reviewed, edited, and verified by the authors for accuracy, originality, and relevance. Generative AI was not used for data collection, statistical analysis, or interpretation of results. No generative AI tool is listed as an author, and the authors accept full responsibility for the scientific and editorial content of the manuscript.

Section 1: Writing and Editing Support

Task: Editing the Summary for grammar and punctuation.
Prompt: “Please review this paragraph for grammar and punctuation. Do not change any text or make other edits, but correct spacing and punctuation errors.”
Output: Returned a grammatically improved version of the paragraph.
Usage: Authors reviewed changes and made additional edits for precision. AI was only used for grammar and punctuation corrections; all interpretation and final revisions were completed by the authors.

Section 2: Formatting and Style Compliance

Task: Formatting references in Harvard style
Prompt: “Reformat the following references to Harvard style as required by Frontiers in Organizational Psychology. Ensure author names, years, titles, and DOIs are correctly aligned.”
Output: Provided a properly formatted reference list.
Usage: Authors manually verified each entry against publisher databases and made necessary corrections. AI support was limited to reference formatting; all substantive decisions about accuracy and style were made by the authors.

Section 3: Summarization for Scoping Literature

Task: Summarizing abstracts for background review
Prompt: “Here are 20 abstracts related to emotional intelligence and organizational performance. Create a table summarizing each abstract with the main research question, method, and findings in no more than 2 sentences each.”

Output: Generated a concise table of summaries.
Usage: Authors cross-checked each summary against the original abstract and edited entries before integrating into the literature review. The abstracts themselves were sourced directly from peer-reviewed journals and databases by the authors. AI produced preliminary summaries, which were reviewed, cross-checked, and substantively revised by the authors.

Section 4: Brainstorming Keywords for Discoverability

Task: Suggesting possible indexing terms
Prompt: "Suggest 15 possible keywords for an article on emotional intelligence decline and organizational wellbeing, aligned with common indexing terms in organizational psychology journals."
Output: Generated a list of candidate keywords.
Usage: AI generated a list of candidate terms; the authors selected and refined keywords that matched the study's scope and journal conventions.

Author Verification
All outputs were reviewed, edited, and verified by the human authors to ensure factual accuracy, relevance, and alignment with the study's intent. No AI tools were used for data analysis, interpretation of results, or drafting of the Introduction or Discussion sections. ChatGPT-5 is not listed as an author.

Model Information
- **Name**: ChatGPT
- **Model:** GPT-5 (ChatGPT, OpenAI)
- **Access period:** June–August 2025
- **Source**: https://chat.openai.com
