# Supplementary material for: The Emotional Recession: global declines in emotional intelligence and its impact on organizational retention, burnout, and workforce resilience
Source: Front Psychol. 2025 Nov 12;16:1701703. doi: 10.3389/fpsyg.2025.1701703 (PMC12646932; doi:10.3389/fpsyg.2025.1701703)
Supplement: Supplementary file 2 [file Data_Sheet_2.PDF]

Supplementary Table S1. Average EQ and Pursuit Noble Goals scores by year, 2019–2024. Annual mean scores for EQ total and the Pursuit of Noble Goals competency.

Supplementary Table S1. Average EQ and Pursuit Scores by Year (2019–2024)

	**2019**	**2020**	**2021**	**2022**	**2023**	**2024**
Total EQ	101.1 (95% CI [100.8, 101.3])	100.2 (95% CI [99.9, 100.4])	99.6 (95% CI [99.3, 99.8])	99.4 (95% CI [99.2, 99.8])	99.1 (95% CI [98.8, 99.4])	99.0 (95% CI [98.5, 99.3])
Know Yourself	101.4 (95% CI [101.0, 101.6])	100.6 (95% CI [100.2, 100.8])	100.0 (95% CI [99.6, 100.3])	99.7 (95% CI [99.4, 100.1])	99.3 (95% CI [98.9, 99.6])	99.0 (95% CI [98.3, 99.4])
Choose Yourself	101.0 (95% CI [100.8, 101.3])	100.2 (95% CI [99.9, 100.4])	99.2 (95% CI [98.9, 99.5])	99.1 (95% CI [99.0, 99.5])	98.8 (95% CI [98.6, 99.1])	98.4 (95% CI [97.8, 98.7])
Give Yourself	100.8 (95% CI [100.5, 101.1])	99.8 (95% CI [99.5, 100.0])	99.5 (95% CI [99.2, 99.8])	99.4 (95% CI [99.2, 99.8])	99.1 (95% CI [98.9, 99.6])	98.5 (95% CI [97.9, 98.9])

Supplementary Table S2. Average SEI competency scores by year, 2019–2024. Annual means for each of the eight emotional intelligence competencies.

Supplementary Table S2. Average Competency Scores by Year (2019–2024)

	2019	2020	2021	2022	2023	2024
**EEL**	100.9 (95% CI [100.6 101.2])	100.1 (95% CI [99.7, 100.4])	99.4 (95% CI [99.1, 99.8])	99.3 (95% CI [98.9, 99.7])	98.8 (95% CI [98.4, 99.2])	98.6 (95% CI [98.0, 99.2])
**RP**	101.8 (95% CI [101.4, 102.1])	100.9 (95% CI [100.6, 101.2])	100.4 (95% CI [100.0, 100.7])	100.2 (95% CI [99.8, 100.6])	99.6 (95% CI [99.2, 100.0])	99.6 (95% CI [99.0, 100.2])
**ACT**	102.1 (95% CI [101.7, 102.4])	102.3 (95% CI [102.0, 102.6])	101.7 (95% CI [101.3, 102.1])	101.0 (95% CI [100.7, 101.4])	100.9 (95% CI [100.6, 101.3])	100.5 (95% CI [99.9, 101.0])
**NE**	98.5 (95% CI [98.1, 98.8])	97.2 (95% CI [96.9, 97.6])	96.5 (95% CI [96.1, 96.9])	96.8 (95% CI [96.4, 97.2])	96.6 (95% CI [96.2, 97.0])	96.5 (95% CI [95.9, 97.1])
**EIM**	101.2 (95% CI [100.9, 101.6])	99.9 (95% CI [99.6, 100.3])	98.8 (95% CI [98.4, 99.2])	99.0 (95% CI [98.6, 99.4])	98.4 (95% CI [98, 98.8])	98.2 (95% CI [97.7, 98.8])
**EO**	102.3 (95% CI [102.0, 102.7])	101.1 (95% CI [100.8, 101.4])	99.9 (95% CI [99.5, 100.3])	100.1 (95% CI [99.7, 100.5])	99.3 (95% CI [98.9, 99.7])	99.3 (95% CI [98.8, 99.9])
**IE**	100.3 (95% CI [99.9, 100.7])	99.3 (95% CI [98.9, 99.6])	99.8 (95% CI [99.4, 100.2])	99.7 (95% CI [99.3, 100.1])	99.6 (95% CI [99.2, 100.0])	98.7 (95% CI [98.1, 99.3])
**PNG**	101.3 (95% CI [101.0, 101.7])	100.2 (95% CI [99.9, 100.6])	99.2 (95% CI [98.8, 99.6])	99.3 (95% CI [98.9, 99.7])	98.7 (95% CI [98.2, 99.1])	98.7 (95% CI [98.0, 99.3])

Supplementary Table S3. Percent change in SEI competencies between 2019 and 2024. Percentage difference in mean scores for each competency across the six-year period.

Supplementary Table S3. Percent change in SEI competencies between 2019 and 2024.

Competency	2019	2024	% change
EEL	100.9 (95% CI [100.6 101.2])	98.6 (95% CI [98.0, 99.2])	-6.46%
RP	101.8 (95% CI [101.4, 102.1])	99.6 (95% CI [99.0, 100.2])	-5.91%
ACT	102.1 (95% CI [101.7, 102.4])	100.5 (95% CI [99.9, 101])	-4.35%
NE	98.5 (95% CI [98.1, 98.8])	96.5 (95% CI [95.9, 97.1])	-5.91%
EIM	101.2 (95% CI [100.9, 101.6])	98.2 (95% CI [97.7, 98.8])	-8.21%
EO	102.3 (95% CI [102.0, 102.7])	99.3 (95% CI [98.8, 99.9])	-8.04%
IE	100.3 (95% CI [99.9, 100.7])	98.7 (95% CI [98.1, 99.3])	-4.52%
PNG	101.3 (95% CI [101, 101.7])	98.7 (95% CI [98, 99.3])	-7.36%

Note: Drive strand competencies are Exercise Optimism, Engage Intrinsic Motivation, Pursue Noble Goals.

Supplementary Table S4. Average SEI Success Factor outcome scores by year, 2019–2024. Annual means for Wellbeing, Effectiveness, Relationships, and Quality of Life.

Supplementary Table S4. Average Outcome Scores by Year (2019–2024)

	2019	2020	2021	2022	2023	2024	% Change (2019–2024)
**Overall Outcomes**	100.9 (95% CI [100.7, 101.1])	100.1 (95% CI [99.8, 100.3])	99.2 (95% CI [99.0, 99.5])	99.1 (95% CI [98.9, 99.4])	99.1 (95% CI [98.8, 99.3])	98.9 (95% CI [98.5, 99.3])	-5.61%
**Effectiveness**	102.3 (95% CI [102.0, 102.7])	101.2 (95% CI [100.9, 101.6])	100.3 (95% CI [100.0, 100.6])	100.4 (95% CI [100.1, 100.8])	100.1 (95% CI [99.8, 100.5])	99.9 (95% CI [99.4, 100.4])	-6.45%
**Relationships**	101.1 (95% CI [100.8, 101.4])	99.7 (95% CI [99.4, 100.0])	99.2 (95% CI [98.8, 99.5])	99.1 (95% CI [98.7, 99.4])	99.3 (95% CI [99.0, 99.7])	98.9 (95% CI [98.4, 99.4])	-6.03%
**Wellbeing**	99.2 (95% CI [98.9, 99.5])	99.0 (95% CI [98.7, 99.3])	98.0 (95% CI [97.7, 98.4])	97.7 (95% CI [97.4, 98.0])	97.4 (95% CI [97.0, 97.7])	97.7 (95% CI [97.2, 98.2])	-4.39%
**Quality of Life**	101.0 (95% CI [100.7, 101.3])	100.1 (95% CI [100.0, 100.6])	99.4 (95% CI [99.0, 99.7])	99.3 (95% CI [98.9, 99.6])	99.4 (95% CI [99.0, 99.7])	99.0 (95% CI [98.5, 99.6])	-5.59%

Supplementary Table S5. Odds ratios for predictive validity of EQ on Success Factors. Forest-plot data showing likelihood of reporting high outcomes (Effectiveness, Relationships, Quality of Life, Wellbeing) for individuals with high EQ.

Supplementary Table S5. Odds Ratios for High EQ and High Success Factors with Confidence Intervals

**Factors**	**Odds Ratio**	**95% Confidence Interval**
Effectiveness	8.34	7.90-8.80
Relationships	5.25	4.99-5.53
Wellbeing	3.63	3.45-3.81
Quality of Life	6.53	6.20-6.88
Overall Outcome	10.18	9.64-10.76
